# Long-term Effectiveness and Adoption of a Cellphone Augmented Reality System on Patients with Stroke: Randomized Controlled Trial

**DOI:** 10.2196/30184

**Published:** 2021-11-23

**Authors:** Chong Li, Xinyu Song, Shugeng Chen, Chuankai Wang, Jieying He, Yongli Zhang, Shuo Xu, Zhijie Yan, Jie Jia, Peter Shull

**Affiliations:** 1 Department of Rehabilitation Medicine Huashan Hospital Fudan University Shanghai China; 2 The State Key Laboratory of Mechanical System and Vibration Shanghai Jiao Tong University Shanghai China; 3 National Clinical Research Center for Aging and Medicine Huashan Hospital Fudan University Shanghai China; 4 National Center for Neurological Disorders Shanghai China

**Keywords:** stroke, augmented reality, serious game, upper limb motor function, cognitive function, home-based rehabilitation

## Abstract

**Background:**

A serious game–based cellphone augmented reality system (CARS) was developed for rehabilitation of stroke survivors, which is portable, convenient, and suitable for self-training.

**Objective:**

This study aims to examine the effectiveness of CARS in improving upper limb motor function and cognitive function of stroke survivors via conducting a long-term randomized controlled trial and analyze the patient’s acceptance of the proposed system.

**Methods:**

A double-blind randomized controlled trial was performed with 30 poststroke, subacute phase patients. All patients in both the experimental group (n=15) and the control group (n=15) performed a 1-hour session of therapy each day, 5 days per week for 2 weeks. Patients in the experimental group received 30 minutes of rehabilitation training with CARS and 30 minutes of conventional occupational therapy (OT) each session, while patients in the control group received conventional OT for the full 1 hour each session. The Fugl-Meyer Assessment of Upper Extremity (FMA-UE) subscale, Action Research Arm Test (ARAT), manual muscle test and Brunnstrom stage were used to assess motor function; the Mini-Mental State Examination, Add VS Sub, and Stroop Game were used to assess cognitive function; and the Barthel index was used to assess activities of daily living before and after the 2-week treatment period. In addition, the User Satisfaction Evaluation Questionnaire was used to reflect the patients’ adoption of the system in the experimental group after the final intervention.

**Results:**

All the assessment scores of the experimental group and control group were significantly improved after intervention. After the intervention. The experimental group’s FMA-UE and ARAT scores increased by 11.47 and 5.86, respectively, and were both significantly higher than the increase of the control group. Similarly, the score of the Add VS Sub and Stroop Game in the experimental group increased by 7.53 and 6.83, respectively, after the intervention, which also represented a higher increase than that in the control group. The evaluation of the adoption of this system had 3 sub-dimensions. In terms of accessibility, the patients reported a mean score of 4.27 (SD 0.704) for the enjoyment of their experience with the system, a mean 4.33 (SD 0.816) for success in using the system, and a mean 4.67 (SD 0.617) for the ability to control the system. In terms of comfort, the patients reported a mean 4.40 (SD 0.737) for the clarity of information provided by the system and a mean 4.40 (SD 0.632) for comfort. In terms of acceptability, the patients reported a mean 4.27 (SD 0.884) for usefulness in their rehabilitation and a mean 4.67 (0.617) in agreeing that CARS is a suitable tool for home-based rehabilitation.

**Conclusions:**

The rehabilitation based on combined CARS and conventional OT was more effective in improving both upper limb motor function and cognitive function than was conventional OT. Due to the low cost and ease of use, CARS is also potentially suitable for home-based rehabilitation.

**Trial Registration:**

Chinese Clinical Trial Registry ChiCTR1800017568; https://tinyurl.com/xbkkyfyz

## Introduction

Stroke is the leading cause of mortality and permanent disability in adults worldwide [1,2]. Upper limb impairment is one of the common consequences after stroke, with up to 80% of survivors experiencing upper limb paresis after stroke onset [3,4]. Furthermore, the decline of cognitive state is present in more than 60% of stroke survivors, typically relevant to degradation of concentration, executive function, and comprehension [5]. Upper limb impairment and decreased cognitive state after a stroke can hamper the activities of the upper limb, which leads to greater dependence in the performance of activities of daily living (ADLs) [6].

The primary mechanism of functional recovery after stroke is synaptic reorganization and neurological functional recovery [7,8], and the bimodal balance-recovery model that links interhemispheric balancing of the brain is the foundation for upper limb function [9]. Studies indicate that high-intensity, repeatable, task-oriented, and feedback rehabilitation training can promote brain remodeling and functional restoration, and enhance the normal bimodal balance-recovery model [10-13]. Clinically, the improvement of upper limb function and cognitive state mainly depends on the treatment of occupational therapists. However, issues such as cost and access may limit the dose of one-on-one rehabilitation exercise with an occupational therapist [14,15]. In addition, patients may have difficulties in keeping concentration when they are doing intensive and repetitive training. Furthermore, stroke survivors still need long-term rehabilitation after being discharged from the hospital. At present, home-based rehabilitation for stroke survivors is mainly based on the exercise prescription provided by therapists. However, patients often abandon rehabilitation because of the boring and repetitive training mode. Additionally, in the progress of rehabilitation, patients can obtain better rehabilitation effects only if they have a high degree of participation. However, treatment methods for self-oriented or home-based rehabilitation are rare. Therefore, convenient rehabilitation systems that can enhance stoke survivors’ enthusiasm and allow them to perform self-guided home-based rehabilitation are needed.

To address these issues, augmented reality (AR) technology has been introduced to the field of stroke rehabilitation. The AR system is a useful new technology that blends virtual objects with real scenes in real time [16]. An increasing number of studies report promising results of its application to stroke rehabilitation [17-19]. At present, the AR systems commonly used in stroke rehabilitation can be divided into conventional AR systems, mirror-based AR systems, and cellphone-based AR systems. However, existing AR systems are not suitable for self-oriented or home-based rehabilitation. Conventional AR systems can enhance patients' motivation, but such systems usually need guidance from therapists [20,21]. Mirror-based AR systems combine AR technology with mirror therapy. Patients can use AR technology for 3D mirror-image treatment, which can effectively promote the recovery of upper limb function. However, this kind of system often requires the patient to wear a head-mounted display or a large device, so patients experience discomfort or difficulty when using it [22]. AR systems based on cellphones have only been used in stroke rehabilitation in recent years. This kind of system is cheaper and more convenient than are other AR technology systems, but few articles have reported on this kind of system for self-guided or home-based rehabilitation. Therefore, AR rehabilitation systems that are suitable for self-oriented home-based treatment are rare and needed.

Based on a review of the relevant literature, we have developed a serious game-based cellphone augmented reality rehabilitation system (CARS). In a previous pilot study performed in a clinical setting, we found that CARS motivated individuals with stroke to perform task-oriented games (eg, Pyramid Reach) during a 30-minute intervention. Most patients who used CARS also reported that the exercise was more motivating than conventional occupational therapy (OT) [23]. An important question is whether or not self-guided exercise with CARS is feasible and improves upper limb function and cognitive state compared with conventional OT in a long-term intervention. In addition, whether this system is suitable for home-based rehabilitation is unknown.

To study these questions, a double-blind randomized controlled trial that compared combined CARS and conventional OT rehabilitation with conventional OT alone was performed. The objective of this paper is twofold: first, to study the long-term effectiveness of the system in improving upper limb function and cognitive state in survivors of subacute stroke; and second, to determine the acceptance of this intervention for home-based rehabilitation of poststroke survivors. We hypothesized that the participants using the combined CARS and conventional OT rehabilitation would receive at least equivalent results to those using conventional OT. We also hypothesized that CARS would be a suitable option for home-based rehabilitation.

## Methods

### Study Design

This was a multicenter, double-blind, 2-group randomized controlled trial comparing combined CARS and conventional OT rehabilitation with matched control conventional OT in patients with upper limb dysfunction in the subacute phase of stroke. The study protocol was approved by the Institutional Review Board of Huashan Hospital, Fudan University (no. KY2018-248) and registered at the Chinese Clinical Trial Registry (ChiCTR1800017568) (Multimedia Appendix 1).

### Serious Game-Based CARS

**Figure 1:**
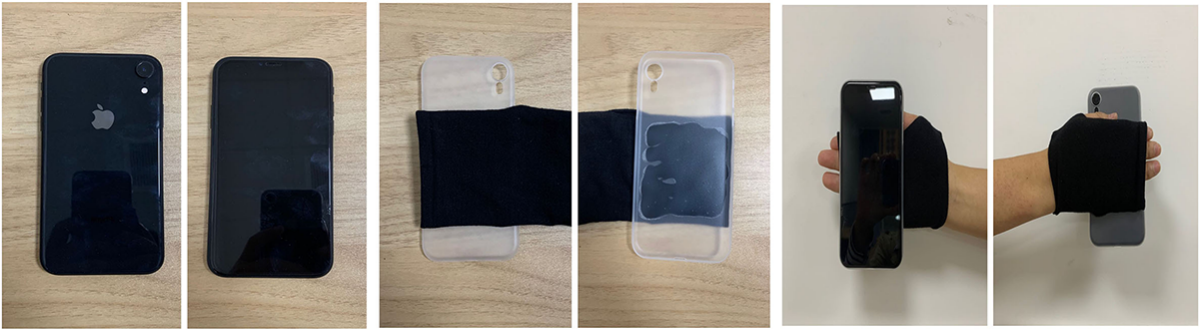
Hardware for the cellphone augmented reality rehabilitation system. (Left) Phone: iPhone XR, iOS 13. (Center) Cellphone cases and gloves. (Right) Method of wearing.

CARS was developed based on the ARKit toolbox run on an iPhone XR cellphone (Apple Inc) with an iOS 13 operating system. Stroke survivors may not be able to pick up the phone due to the weakness of their affected side. Therefore, a fingerless glove cellphone case was designed, which could easily fix the cellphone to the patient’s affected hand. Patients could move their affected upper limb to use the cellphone and interact with 3D virtual targets generated on the cellphone screen (Figure 1).

**Figure 2:**
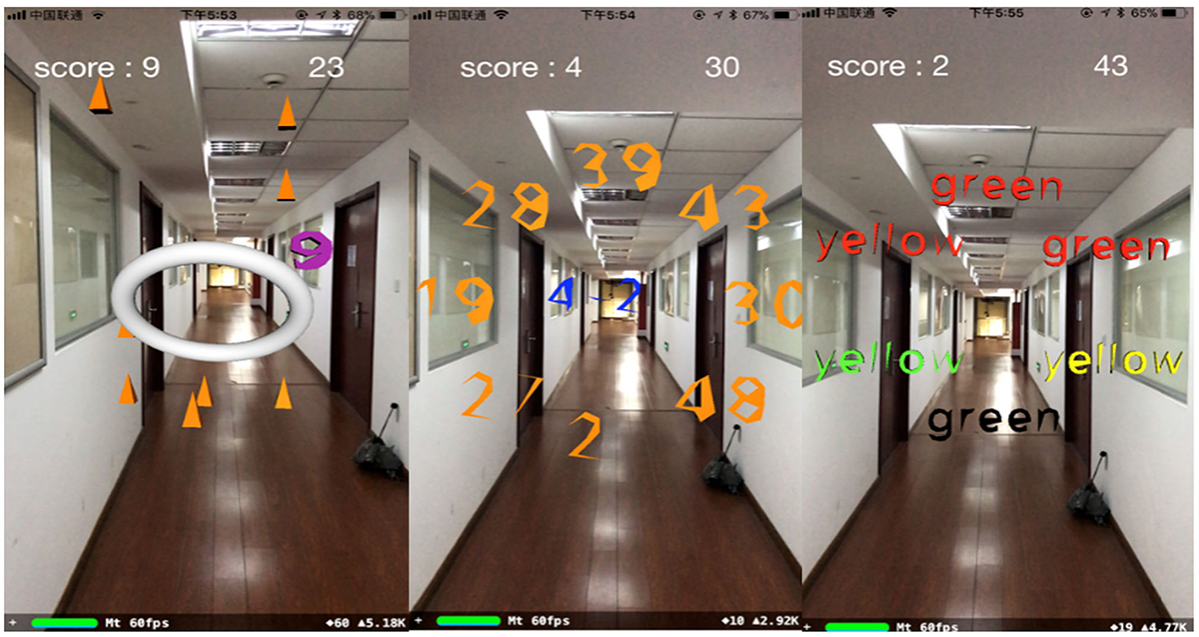
Three augmented reality–based serious games for rehabilitation of upper limb motor function and cognitive function. (Left) Pyramid Reach. (Center) Add VS Sub. (Right) Stroop Game.

Three serious games were developed based on CARS for improving both motor function and cognition function of stroke survivors (Figure 2) [23]. The first game is Pyramid Reach, which was designed to enhance upper limb function and concentration of stroke survivors. In this game, patients are expected to move the cellphone to touch the virtual pyramid targets. This is the basis of an engaging game to help patients reach out for virtual targets in a certain period of time. The second game is Add VS Sub (AVS), which was intended to strengthen both physical and cognitive ability. In this game, patients need to calculate the formula generated in the center of the screen and touch the correct answer in the set of numbers that appear around the formula. The third game, Stroop Game (SG), aims to train both motor and cognitive function. SG is based on the Stroop effect which is our tendency to experience difficulty naming a physical color when it is used to spell the name of a different color. These 3 serious games have the potential to keep patients engaged and motivated even though they are performing continuous and repetitive movements.

The proposed system can record the scores of each round and the trajectory of user’s upper limb distal during the training, which enables clinicians to track the change in the range of motion of the patient’s affected side and the progress of the patient’s recovery. This function can help clinicians give further diagnosis and rehabilitation guidance to outpatients.

### Participants

Thirty participants were recruited in the study between August 2020 and March 2021. The inclusion criteria were the following: age ≥20 and <70 years; first incidence of a stroke with unilateral hemiparesis; chronicity ≥ 7 and＜180 days, Mini-Mental State Examination (MMSE) score ≥20, Brunnstrom stage for upper limb ≥3, ability to give informed consent and operate a mobile phone, and the visual and mental ability to actively participate in the protocol. Exclusion criteria were the following: history of epilepsy orthopedic alteration or pain syndrome of the upper limb, severe aphasia or other psychiatric illnesses that limit the ability to participate or give consent, visual disturbance such as visuospatial neglect, and poor sitting balance.

PASS software (NCSS Statistical Software) was used to calculate the required sample size. The Fugl-Meyer Assessment for Upper Extremity (FMA-UE) subscale was considered significant when the change value was more than 5 points [24]. To satisfy an α level of .05 and a power of 0.95, a minimum of 12 patients was required in each of the 2 groups. Assuming a dropout rate of 20%, we aimed to include 15 patients in each of the 2 groups.

### Study Procedures and Interventions

All participants were invited for an initial assessment to confirm that they met the inclusion criteria. An independent researcher not involved in the study created a blocked randomization sequence using a computerized program (Microsoft Excel). Block randomization ensured equal numbers of participants for the experimental and the control group. Allocation assignments were placed in sequentially numbered, opaque, and sealed envelopes by an offsite officer not involved in the study. Patients were not blind regarding the intervention received. Once the participant passed the screening process and completed the baseline assessment, an independent researcher would open an envelope and reveal the group allocation. In addition, outcome evaluators were blinded to the group assignment.

After giving informed consent, eligible participants were allocated to 1 of 2 groups. A previous study by Saposnik et al [25] had shown that eight 60-minute sessions of Wii gaming over 2 weeks in a group of subacute stroke survivors resulted in significant improvements. Therefore, participants were instructed to train for 10 hours, divided into 5 days per week for 2 weeks. In both groups, the total treatment dose was matched at 1 hour per day. Patients allocated to the experimental group were treated for 30 minutes using CARS and 30 minutes of conventional OT per day. The 30 minutes CARS therapy consisted of 15 trails, 5 trials per game. The patients were monitored by therapists or their caretakers when they were using the system. If there was an adverse event, the therapist or clinician would examine the patient and deal with it promptly. In addition, the therapists or clinicians would record the adverse event. Patients in the control group received conventional OT for 1 hour each day. Conventional OT consisted of passive and active range of motion exercises, muscle strengthening, and functional tasks that matched CARS therapy. The occupational therapists selected relevant motor and cognitive training according to the patient's functional status.

**Figure 3:**
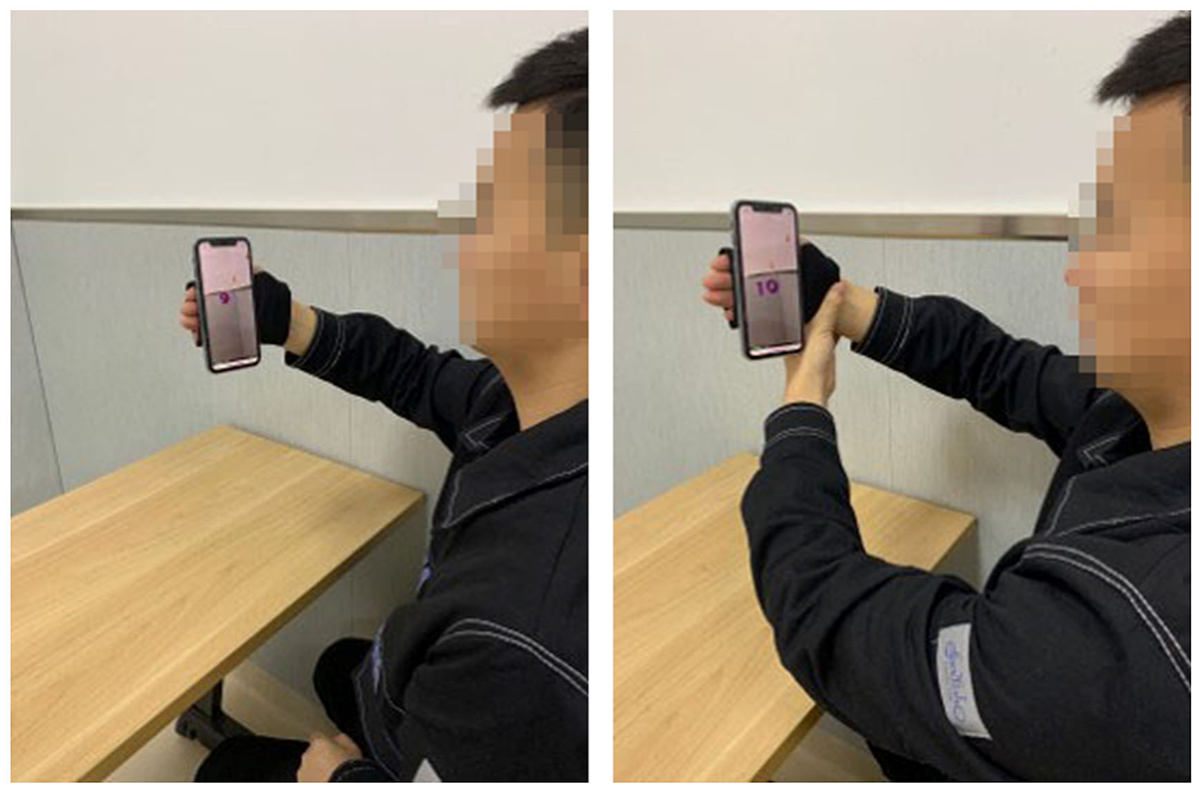
Two training methods. (Left) The participant training individually with the affected side. (Right) The participant using the unaffected side to assist the affected side for training.

For the experimental group, we provided 2 methods for patients to use the system (Figure 3). We encouraged patients to use their affected hand to play serious games. However, if the patients had upper limb motor function, they could use the unaffected side to assist the affected side to perform movements and interact with the targets generated from the serious games. Before the intervention, patients in the experimental group were instructed on how to use the serious game–based cellphone AR system. Patients moved their hands back and forth in front of them for 5 seconds to initialize the system and allow the cellphone to familiarize itself with the surrounding environment; they then performed a practice trial for each game to become familiar with the game function and operation.

### Outcome Measures

#### Motor Function Assessment

Four effective clinical-based assessments were selected and performed to evaluate the upper limb motor function of patients in both groups before and after the intervention. Primary outcomes were the FMA-UE and the Action Research Arm Test (ARAT). The FMA-UE can objectively measure arm impairment and the degree of muscle synergies present. Performance is rated on a 3-point ordinal scale from 0 to 2 with a maximum score of 66. A higher score indicates minimal or no impairment [26-28]. The internal consistency and validity are excellent [29,30]. The ARAT can objectively assess arm and hand function using observational methods. The ARAT is divided into 4 subtests of grasp, grip, pinch, and gross arm movement. Performance on each item is rated on a 4-point ordinal scale from 0 to 3 with a maximum score of 57, with a higher score indicating a better level of function [31]. The reliability and validity are excellent [32,33].

In addition, the manual muscle test (MMT) and Brunnstrom stage (BS) were selected as second motor outcomes. The MMT is a procedure for the evaluation of muscle strength, based on the effective performance of a movement in relation to the forces of gravity or manual resistance through the available range of motion. The grading of MMT ranges from 0 (no visible or palpable contraction) to 5 (full range of motion against gravity, maximal resistance) [34]. The observational cohort studies indicated good internal and external validity of the MMT [35]. BS is used by therapists to assess how well their patients are recovering. The stages of BS range from 1 (flaccid paralysis) to 6 (normal function) [36,37]. 

#### Cognition Function Assessment

To assess the change of cognitive function for patients in both the experimental and control groups, 1 clinical-based assessment and 2 cognition evaluations designed based on the proposed serious games were performed additionally before and after the intervention.

MMSE is a widely used test of cognitive function for stroke survivors and includes tests of orientation, attention, memory, language, and visual-spatial skills [38]. A higher score indicates a better level of cognitive function, with a maximum score of 30. The reliability and validity are excellent [39-41].

Game 2 (AVS) and game 3 (SG) can train the patient's ability of calculation and comprehension. The scores of the 2 games trained by patients each day were recorded by CARS, and these scores were derived and analyzed. We further designed a paper version of AVS and SG to test the change of cognitive ability in patients. The patients were asked to answer the questions from the paper version as quickly as possible within 1 minute. The number of correct answers was the score of the patients.

#### ADL Assessment

The Barthel index (BI) is used to measure performance in ADLs. Ten variables describing ADL and mobility are scored, with a higher number being a reflection of greater ability to function independently following hospital discharge. Scores of 0 to 20 indicate “total” dependency, 21 to 60 indicate “severe” dependency, 61 to 90 indicate “moderate” dependency, and 91 to 99 indicate “slight” dependency [42-44]. The reliability and validity are good [45].

#### Questionnaire

Referring to the User Satisfaction Evaluation Questionnaire [46], we provided a 7-question questionnaire that assayed the patient’s perceived engagement and the acceptability of their experience at the end of therapy. This questionnaire assessed the patient’s feelings regarding the gaming experience, their perception of comfort, and their enjoyment of the game (1, strongly disagree, 2, disagree; 3, neutral; 4, agree; 5, strongly agree). We also asked an additional 2 questions about their previous experience and their suggestions about the system: (1) Do you experience any negative symptoms during or after gameplay? (2) What are some suggestions you have that you think could improve the system?

### Statistical Analysis

Statistical analysis was performed in SPSS version 26 (IBM Corp). Data were confirmed to have a normal distribution according to the Shapiro-Wilk normality test since the sample size was small. Two-tailed *t* tests (for continuous variables) and the chi-square test (for categorical variables) were used to compare baseline measures between the 2 treatment groups. Nonparametric tests were used if the data seemed to be nonnormally distributed. The Wilcoxon signed rank test was used for within-group analyses, and the Mann-Whitney test was used for between-group analyses. The level of statistical significance was set at a *P* value <.05. Intention to treat was applied, and missing data were replaced by the mean of the previous outcomes of the given patient.

## Results

### Participant Characteristics

**Figure 4:**
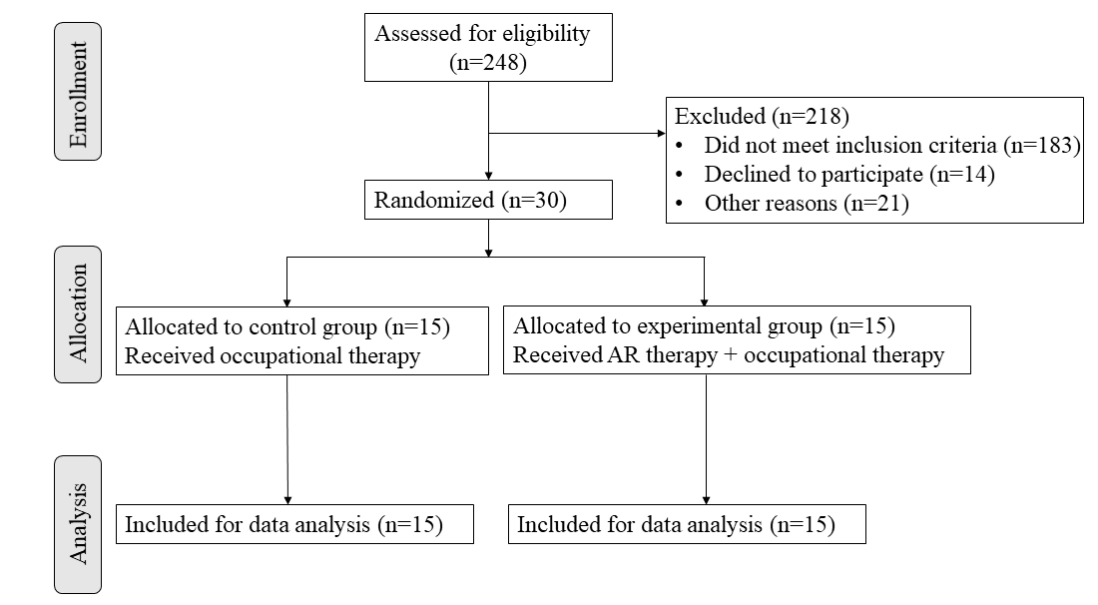
Flowchart for participant selection and assignment. AR: augmented reality.

The flowchart for participants is presented in Figure 4. Initially, 248 hospitalized patients in Huashan Hospital, Nanshi Hospital affiliated to Henan University, and The Third Rehabilitation Hospital of Shanghai were assessed for eligibility, but 218 patients did not meet the inclusion criteria. Finally, 30 patients were enrolled and randomly divided into 2 groups: 15 patients were allocated to the experimental group and 15 patients were allocated to the control group. None of the participants withdrew from the study.

The patients’ baseline demographic and clinical characteristics are shown in Table 1. The time from onset ranged from 9 to 160 days, with an average of 73.3 days. Furthermore, 63% (19/30) of the patients were men, and their age ranged from 26 to 75 years, with an average age of 53.07 years. No significant differences were observed between the groups regarding gender, age, duration after stroke onset, stroke type, or affected side. There were no significant differences in the baseline values of the motor outcomes between the 2 groups (FMA-UE: *P*=.56; ARAT: *P*=.68; MMT shoulder: *P*=.81; MMT elbow: *P*=.53; MMT wrist: *P*=.20). The baseline values of the cognitive outcomes (MMSE: *P*=.87; AVS: *P*=41; SG: *P*=.53) were well distributed with no significant between-group differences.

**Table 1:** Baseline demographic and clinical characteristics of the patients.

Variable		Experimental group (N=15)	Control group (N=15)	*t* or *z* value		*P* value	
Age (years), median (IQR)		62 (24)	57 (32)	0.062^a^		.96	
**Gender, n (%)**					0.144^b^		.70
	Male	10 (66)	9 (60)				
	Female	5 (33)	6 (40)				
Time from onset (days), mean (SD)		78.2 (40)	69.2 (51)	0.530^c^		0.60	
**Affected side, n (%)**					0.133^b^		.71
	Left	8 (53)	7 (46)				
	Right	7 (46)	8 (53)				
MMSE^d^, median (IQR)		26 (4)	25 (3)	0.168^a^		.87	
FMA-UE^e^, median (IQR)		30 (18)	25 (23)	0.583^a^		.56	
ARAT^f^, median (IQR)		14 (17)	12 (22)	0.417^a^		.68	
BS-U^g^, median (IQR)		3 (1)	3 (1)	0.024^a^		.99	
BS-H^g^, median (IQR)		3 (2)	4 (3)	0.085^a^		.93	
MMT^h^ shoulder, median (IQR)		3 (0)	3 (0)	0.338^a^		.81	
MMT elbow, median (IQR)		3 (0)	3 (1)	0.898^a^		.53	
MMT (wrist), median (IQR)		1 (3)	3 (1)	1.346^a^		.20	
BI^i^, mean (SD)		64.67 (12)	63 (13)	0.354^c^		.72	
AVS^j^, mean (SD)		17.8 (4)	19.47 (6)	0.841^c^		.41	
SG^k^, mean (SD)		11.67 (5)	13 (6)	0.625^c^		.53	

^a^Wilcoxon rank sum test.

^b^Chi-square test.

^c^Two-tailed *t* test.

^d^MMSE: Mini-Mental State Examination.

^e^FMA-UE: Fugl-Meyer Assessment of the Upper Extremity.

^f^ARAT: Action Research Arm Test.

^g^BS: Brunnstrom stage (U: upper extremity; H: hand).

^h^MMT: manual muscle test.

^i^BI: Barthel index.

^j^AVS: Add VS Sub.

^k^SG: Stroop Game.

### Comparison of Motor Function

**Figure 5:**
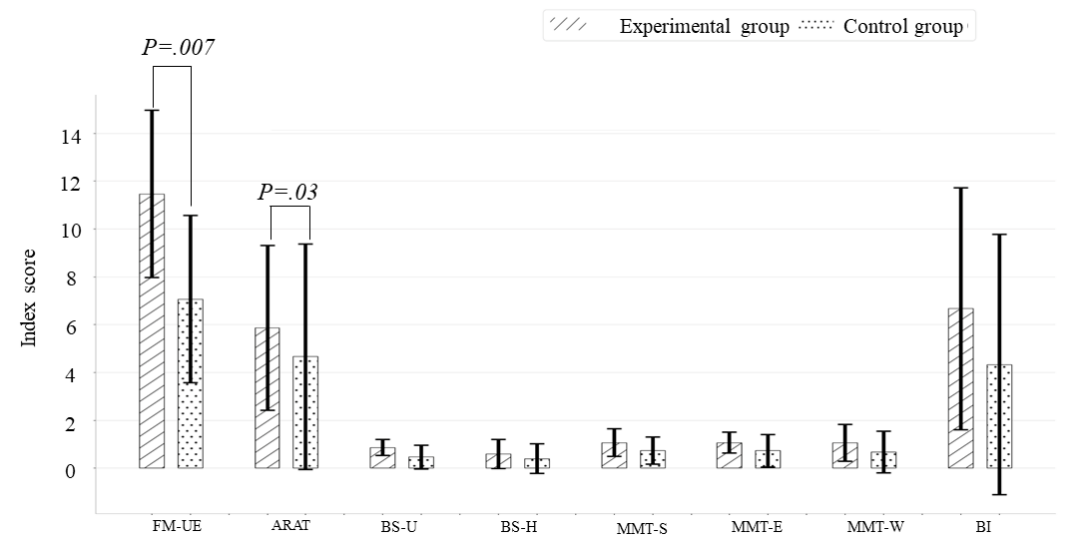
Longitudinal changes in motor outcomes with the experimental group showing significantly greater improvements than the control group in FMA-UE and ARAT. ARAT: Action Research Arm Test; BI: Barthel index; BS: Brunnstrom stage (U: upper extremity; H: hand); FMA-UE: Fugl-Meyer Assessment of the Upper Extremity; MMT: manual muscle test (S: shoulder; E: elbow; W: wrist).

After the intervention, both groups showed significant improvement in the FMA-UE, ARAT, BS, and MMT scores over time (Table 2). The experimental group’s score of FMA-UE and ARAT increased by 11.47 and 5.86, respectively, after intervention, and were both significantly higher than the corresponding increase ih the control group (Figure 5). Additionally, a considerable change in Brunnstrom stage-hand (mean 0.60) and MMT wrist (mean 1.07) in the experimental group was found compared to the control group. Other than this, there were no between-group differences in terms of Brunnstrom stage-upper extremity, MMT shoulder, MMT elbow, or BI.

**Table 2:** Comparison of outcomes in the experimental group and control group.

Outcomes	Experimental group (N=15)				Control group (N=15)		
	Pretest	Posttest	*P* value	Pretest		Posttest	*P* value
FMA-UE^a^, median (IQR)	30 (18)	41 (21)	.001	25 (23)		33 (26)	.001
ARAT^b^, median (IQR)	14 (17)	21 (23)	.001	12 (22)		16 (24)	.001
MMSE^c^, median (IQR)	26 (4)	27 (2)	.005	25 (5)		25 (4)	.004
BS-U^d^, median (IQR)	3 (1)	4 (1)	﹤.001	3 (1)		4 (2)	.008
BS-H^d^, median (IQR)	3 (2)	4 (3)	.007	4 (3)		4 (3)	.034
MMT^e^ shoulder, median (IQR)	3 (0)	4 (1)	.001	3 (0)		4 (0)	.002
MMT elbow, median (IQR)	3 (0)	4 (0)	﹤.001	3 (1)		4 (0)	.005
MMT wrist, median (IQR)	1 (3)	3 (2)	.001	3 (1)		3 (2)	.014
BI^f^, median (IQR)	65 (25)	75 (15)	.002	60 (25)		65 (20)	.01
AVS^g^, median (IQR)	17 (4)	25 (5)	.001	18 (10)		23 (10)	.001
SG^h^, median (IQR)	10 (6)	18 (10)	.001	12 (7)		15 (8)	.001

^a^FMA-UE: Fugl-Meyer Assessment of the Upper Extremity.

^b^ARAT: Action Research Arm Test.

^c^MMSE: Mini-Mental State Examination.

^d^BS: Brunnstrom stage (U: upper extremity; H: hand).

^e^MMT: manual muscle test.

^f^BI: Barthel index.

^g^AVS: Add VS Sub.

^h^SG: Stroop Game.

### Comparison of Cognitive Function

**Figure 6:**
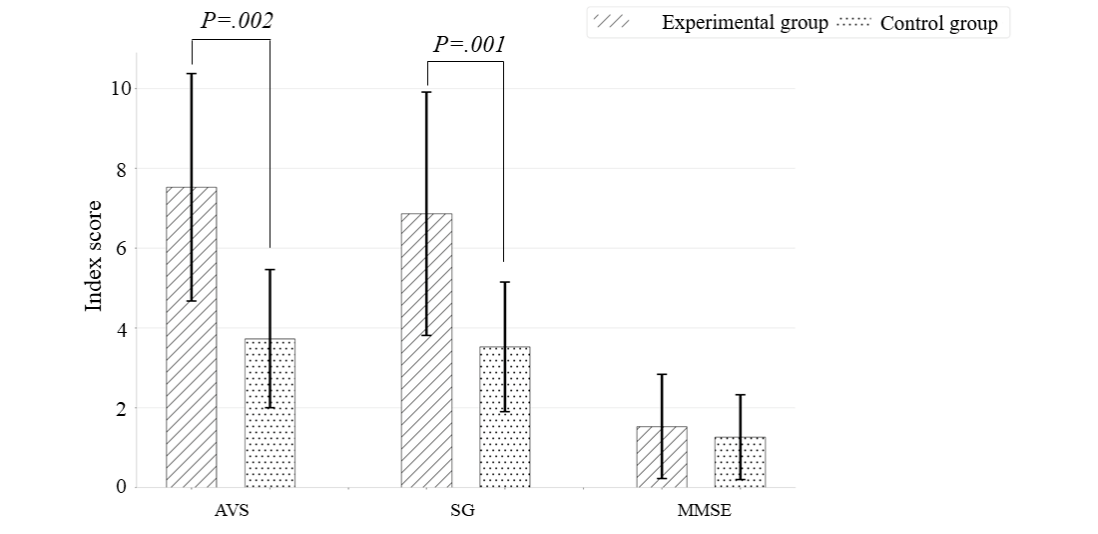
Longitudinal changes in Add VS Sub (AVS), Stroop Game (SG), and Mini-Mental State Examination (MMSE) with the experimental group showing significantly greater improvements than the control group in AVS and SG. AVS: Add VS Sub; MMSE: Mini-Mental State Examination; SG: Stroop Game.

Significant group interaction effects were observed for AVS and SG scores (Table 2). The average AVS and SG score in the experimental group increased to 7.53 and 6.83, respectively, after intervention, which was statistically significant compared to the increase in the control group (Figure 6). However, no significant between-group difference was observed in the MMSE.

**Figure 7:**
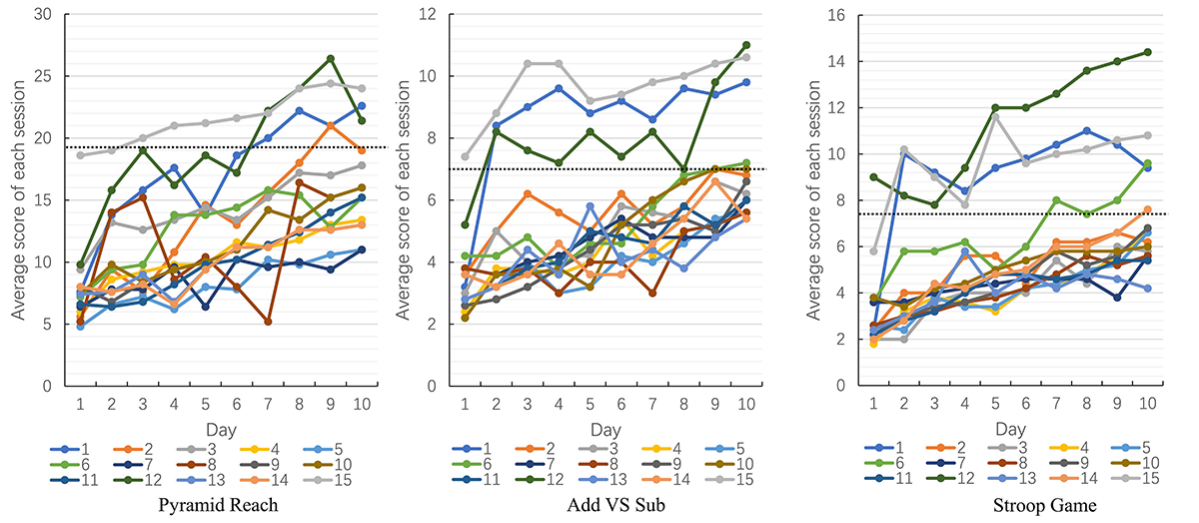
Line charts of 15 patients’ daily scores for 3 serious games. The average score of 5 trails represents a session, and each color represents a patient.

We derived all patients’ training scores recorded on the mobile phone. After processing and analyzing the scores, we created a line chart of each patient (Figure 7). Overall, the final mean score of 3 serious games for each patient improved to some extent compared to the first mean score.

### Clinical Acceptability

A user satisfaction questionnaire was administered for patients at the end of their final intervention in the experimental group. The 5-point rating was used to assess responses to the questions (1, strongly disagree; 2, disagree; 3, neutral; 4, agree; 5, strongly agree). In terms of accessibility, the patients reported a mean score of 4.27 (SD 0.704) for the enjoyment of their experience with the system, a mean 4.33 (SD 0.816) for success in using the system, and a mean 4.67 (SD 0.617) for the ability to control the system. In terms of comfort, the patients reported a mean 4.40 (SD 0.737) for the clarity of information provided by the system and a mean 4.40 (SD 0.632) for comfort. In terms of acceptability, the patients reported a mean 4.27 (SD 0.884) for usefulness in their rehabilitation (Table 3). All patients in the experimental group thought that CARS can be a good option for self-oriented or home-based rehabilitation.

Here, we report patients’ suggestions for the system. Some patients felt that the cellphone screen was too small to see clearly. Some patients thought these 3 AR serious games should be set to a different level, so that they can be applied to patients in different stages.

**Table 3:** Results of the acceptability questionnaire^a^.

Questions	Score, mean (SD)
Q1. Did you enjoy your experience with the system?	4.27 (0.704)
Q2. Were you successful using the system?	4.33 (0.816)
Q3. Were you able to control the system?	4.67 (0.617)
Q4. Is the information provided by the system clear?	4.40 (0.737)
Q5. Did you feel comfortable during your experience with the system?	4.40 (0.632)
Q6. Do you think that this system will be helpful for your rehabilitation?	4.27 (0.884)
Q7. Do you think this system can be used for home-based rehabilitation?	4.67 (0.617)

^a^The questionnaire includes 7 questions, each with a score of 1 to 5 (1, strongly disagree; 2, disagree; 3, neutral; 4, agree; 5, strongly agree).

### Safety

No significant adverse events related to CARS occurred during the clinical research. Reported adverse events were pain and fatigue in the shoulder (n=3) and elbow (n=2), which were foreseen and already mentioned to the patients before therapy began. The use of this cellphone game-based AR system was safe and acceptable for all participants in the study.

## Discussion

### Principal Results

We have developed a serious game–based cellphone augmented reality rehabilitation system for upper limb recovery and improving cognitive function after stroke. Compared with conventional OT, combined CARS and conventional OT rehabilitation proved to be more effective in improving both upper limb function and cognitive function. As a new rehabilitation technique, CARS can replace part of traditional OT and be used as a supplementary treatment method in the hospital to decrease the consumption of medical resources and reduce the burden of patients. In addition, this system can be a suitable option for self-oriented or home-based rehabilitation.

Our findings showed that CARS effectively enhanced upper extremity recovery in patients with stroke. In the comparison between CARS and conventional OT, intervention using CARS showed greater improvement in the FMA-UE and ARAT scores. Comparable improvements between groups were shown in the BS, MMT, and BI. Our results showed that compared with the control group, the experiment group using CARS experienced effectively improved calculation and color-matching ability. In addition, patients in the experimental group completed the 2-week intervention using CARS without severe adverse effects, and they were satisfied with this system for self-oriented or home-based rehabilitation. We speculated that the therapeutic effectiveness of combined CARS and conventional OT rehabilitation would be equal to or greater than that of conventional OT alone, and the results confirmed our hypothesis. The results that CARS can effectively improve the upper limb function and cognitive state of patients may be related to the following factors. Studies conclude that serious games seem to be a safe rehabilitation modality for patients recovering from stroke [47]. The 3 AR serious games we designed can provide immediate feedback, enjoyment, high engagement, and task-oriented movement. Additionally, the AVS game and SG were specially designed for training calculation and color-matching ability. Moreover, visual and haptic feedback can enhance the patients’ desire for interaction. Therefore, CARS can facilitate motor learning, improve cognition function, and increase rehabilitation motivation.

Although some studies have attempted to use AR-based rehabilitation systems for patients with stroke, few studies have included a control group. Assis et al [48] developed a system based on AR for upper-limb motor rehabilitation of stroke survivors. Two case studies were conducted to determine the clinical feasibility. Hoermann et al [49] presented a novel AR system in combination with a validated mirror therapy for stroke survivors. The study examined the therapeutic intervention in 5 patients. Kaneko et al [50] developed a novel approach using AR that could improve self-body cognition in stroke survivors. The study clarified the effect of the system by treating 11 patients. However, none of these studies included a control group. Additionally, most studies that compared AR therapy with conventional therapy allowed more therapy time in the experimental group [51,52]. Consequently, we are not sure whether these results indicate the AR system’s greater effectiveness or are rather due to the effect of excess treatment time. Moreover, few studies have determined the acceptance for self-oriented home-based rehabilitation, and many studies have focused on only a single motor aspect rather than on multiple aspects. Therefore, we tried to design a multiaspect (motor function, cognitive function, and acceptance) randomized controlled trial with a matched intervention dose.

Compared with other systems, the proposed CARS we developed has the following advantages. First, our system requires the least amount of equipment and is also the cheapest and most convenient. CARS was developed for users with a mobile phone because almost everyone has one. The system is portable and very easy to use regardless of a person’s location. Other AR systems often require independent 3D tracking systems, monitors, and interactive systems, and we integrate all 3 into a single cellphone [53,54]. Second, we use dual-task training to simultaneously train patients' motor and cognitive abilities. In addition, we adopted the training theory involving central-peripheral-central closed-loop training [55]. Patients start task training by activating the central nervous system of consciousness. When the patient completes the task, the peripheral cellphone gives the patient a short vibration stimulus, which uploads and then strengthens the central control function of the patient.

### Limitations

There are a few limitations in this study. First, although compensatory movements were restricted during the intervention, they were not controlled during the assessment, which could have influenced the performance in the scales and tests. Second, although the physical therapist who assessed the participants’ condition did not know the protocol, the therapists who administered and controlled the intervention were not blind. Third, we use gloves of uniform size, but each patient had a different hand size, and thus it might have been difficult for some patients to wear the gloves. Fourth, none of the patients who participated in the study had any experience with AR rehabilitation. Therefore, they could not compare our games with other similar games. Finally, the sample of the study (N=30) can be considered small, which may limit the degree to which the results can be extrapolated.

### Future Studies

In future research, we will further improve our devices, which will involve developing an Android version to reduce installation costs, preparing different specifications of gloves for patients with different hand sizes, and increasing the variety and difficulty settings of the game. In addition, future work will include more engaging serious games to increase the variety of therapy solutions and adaptability to patient abilities so that a therapist or patient can match the degree of challenge necessary to keep the rehabilitation advancing. In the trial design, large-sample, controlled, follow-up clinical studies will be conducted in the future to verify the long-term efficacy of the system for home-based rehabilitation.

### Conclusions

At the behavioral level, there was additional benefit received from CARS. Combined CARS and conventional OT rehabilitation was more effective in improving both upper limb function and cognitive function compared with conventional OT alone. The results of our study indicated that the proposed CARS can replace the one-on-one conventional OT delivered by an occupational therapist and that the system can be used as an assistant therapeutic tool in the hospital. In addition, CARS is convenient, low-cost, and user-friendly, which indicates that this system is also suitable for home-based rehabilitation. Future studies with a longer intervention time and a follow-up of patients for home-based rehabilitation are needed to explore the effectiveness of the system.

## Appendix

Multimedia Appendix 1 Consolidated Standards of Reporting Trials (CONSORT) eHealth checklist (v 1.6.1).

## Abbreviations

ADL: activity of daily living
ARAT: Action Research Arm Test
AVS: Add VS Sub
BI: Barthel index
BS: Brunnstrom stage
CARS: cellphone augmented reality system
FMA-UE: Fugl-Meyer Assessment Upper Extremity
MMSE: Mini-Mental State Examination
MMT: manual muscle test
SG: Stroop Game
